# Supplementary material for: Striatal dopamine can enhance both fast working memory, and slow reinforcement learning, while reducing implicit effort cost sensitivity
Source: Nat Commun. 2025 Jul 9;16:6320. doi: 10.1038/s41467-025-61099-0 (PMC12241355; doi:10.1038/s41467-025-61099-0)
Supplement: Supplementary file 1 — Supplementary Information [file 41467_2025_61099_MOESM1_ESM.pdf]

Supplementary Tables

Bayesian mixed effects model of training phase accuracy

The following mixed effects logistic regression was fitted to trial wise accuracy across all participants and drug conditions. All effects had both random and fixed effects terms. The model was fit using the *brms* package version 2.8.0 in R. Note that the logistic regression model is fit based on delay modeled as $-1/n_d$ where n_d is the number of trials since the last previous correct iteration, and all numeric predictors are z-scored.

Supplementary Table S1

	Trial-wise Training Phase Accuracy		
Predictors	*Log-Odds*	*CI*	*P*
Intercept	3.56	3.42 – 3.72	**$<2.1\times10^{-16}$**
Set Size (n_s)	-0.15	-0.24 – -0.06	**1.1×10^{-3}**
Delay (n_d)	-0.05	-0.14 – 0.04	0.75
DA Synth. Capacity (DA)	0.17	0.02 – 0.32	**0.026**
Methylphenidate (MPH)	0.41	0.24 – 0.57	**1.1×10^{-6}**
Sulpiride (SUL)	-0.27	-0.41 – -0.13	**1.5×10^{-4}**
Previous Correct Iterations (pCor)	0.58	0.49 – 0.67	**$<2.1\times10^{-16}$**
Session Number (Sess)	0.10	0.04 – 0.15	**3.6×10^{-4}**
n_s * n_d	-0.28	-0.35 – -0.21	**4.4×10^{-15}**
n_s * DA	-0.10	-0.019 – -0.01	**0.029**
n_d * DA	0.04	-0.04 – 0.13	0.35
n_s * MPH	0.02	-0.11 – 0.14	0.75
n_s * SUL	0.03	-0.08 – 0.14	0.65
n_d * MPH	-0.07	-0.20 – 0.06	0.30
n_d * SUL	-0.11	-0.23 – -0.00	**0.047**
DA * MPH	-0.07	-0.23 – 0.09	0.40
DA * SUL	-0.01	-0.15 – 0.13	0.90
n_s * pCor	0.16	0.08 – 0.24	**8.9×10^{-5}**
DA * pCor	-0.01	-0.10 – 0.08	0.84
MPH * pCor	0.20	0.06 – 0.33	**0.004**
SUL * pCor	-0.04	-0.15 – 0.07	0.49

n_d * pCor	0.15	0.08 – 0.23	**8.9×10^{-5}**
n_s * n_d	-0.28	-0.35 – -0.21	**4.4×10^{-15}**
n_s * n_d * DA	-0.08	-0.14 – -0.01	**0.016**
n_s * n_d * MPH	-0.02	-0.12 – 0.09	0.72
n_s * n_d * SUL	-0.01	-0.11 – 0.08	0.85
n_s * DA * MPH	0.03	-0.10 – 0.15	0.65
n_s * DA * SUL	0.06	-0.04 – 0.17	0.27
n_d * DA * MPH	-0.08	-0.21 – 0.04	0.21
n_d * DA * SUL	-0.02	-0.13 – 0.09	0.73
n_s * DA * pCor	0.03	-0.05 – 0.11	0.47
n_s * MPH * pCor	-0.04	-0.17 – 0.09	0.56
n_s * SUL * pCor	-0.06	-0.17 – 0.05	0.29
DA * MPH * pCor	0.02	-0.11 – 0.15	0.78
DA * SUL * pCor	0.01	-0.09 – 0.11	0.86
n_d * DA * pCor	-0.06	-0.13 – 0.02	0.90
n_d * MPH * pCor	0.07	-0.05 – 0.20	0.76
n_d * SUL * pCor	-0.00	-0.11 – 0.10	0.99
DA * MPH * pCor	0.02	-0.11 – 0.15	0.78
DA * SUL * pCor	0.01	-0.09 – 0.11	0.86
n_d * DA * pCor	-0.06	-0.13 – 0.02	0.90
n_d * MPH * pCor	0.07	-0.05 – 0.20	0.76
n_d * SUL * pCor	-0.00	-0.11 – 0.10	0.99
n_s * n_d * DA * MPH	0.05	-0.05 – 0.15	0.33
n_s * n_d * DA * SUL	0.10	0.01 – 0.19	**0.029**
n_s * DA * MPH * pCor	-0.02	-0.15 – 0.10	0.77
n_s * DA * SUL * pCor	-0.05	-0.15 – 0.06	0.36
n_d * DA * MPH * pCor	0.06	-0.06 – 0.18	0.33
n_d * DA * SUL * pCor	0.11	0.00 – 0.21	**0.036**
N_{id}	92		
Observations	77699		

Bayesian mixed effects model of test phase accuracy

The following mixed effects logistic regression was fitted to trial wise selection of the stimulus which was rewarded at the higher rate in the test phase across all participants and drug conditions. All effects had both random and fixed effects terms. The model was fit using the *brms* package version 2.8.0 in R. Note that the logistic regression model is fit to trials with reaction times slower than 0.25 seconds, and all numeric predictors are z-scored.

Supplementary Table S2

	Trial-wise Testing Phase Accuracy		
Predictors	*Log-Odds*	*CI*	*p*
Intercept	0.08	0.02 – 0.14	**0.009**
Value Difference (ΔV)	0.31	0.23 – 0.38	**4.4×10^{-16}**
Mean Set Size ($\bar{n}_s$)	0.03	-0.01 – 0.08	0.19
Mean Value ($\bar{V}$)	-0.00	-0.05 – 0.05	0.97
DA Synth. Capacity (DA)	0.01	-0.05 – 0.08	0.78
Methylphenidate (MPH)	-0.01	-0.09 – 0.06	0.81
Sulpiride (SUL)	-0.05	-0.13 – 0.02	0.19
Set Size Difference (Δn_s)	-0.28	-0.38 – -0.18	**4.1×10^{-8}**
Trial Number	0.03	-0.00 – 0.06	0.057
Session Number	0.01	-0.02 – 0.05	0.59
$\Delta V * \bar{n}_s$	0.06	0.00 – 0.11	**0.028**
$\Delta V * \bar{V}$	-0.01	-0.09 – 0.08	0.83
ΔV * DA	0.02	-0.06 – 0.10	0.14
$\bar{n}_s$ * DA	-0.01	-0.06 – 0.05	0.73
$\bar{V}$ * DA	-0.04	-0.10 – 0.02	0.19
ΔV * MPH	0.09	-0.05 – 0.23	0.21
ΔV * SUL	0.02	-0.09 – 0.14	0.75
$\bar{n}_s$ * MPH	-0.01	-0.08 – 0.06	0.79
$\bar{n}_s$ * SUL	-0.01	-0.08 – 0.05	0.78
DA * MPH	0.03	-0.05 – 0.11	0.47
DA * SUL	-0.02	-0.10 – 0.07	0.66
$\bar{V} * \Delta n_s$	0.01	-0.06 – 0.08	0.79

DA * Δn_s	-0.01	-0.13 – 0.06	0.85
MPH * Δn_s	0.12	0.01 – 0.22	**0.025**
SUL * Δn_s	0.03	-0.06 – 0.12	0.52
ΔV * $\bar{n}_s$ * $\bar{V}$	0.04	-0.02 – 0.10	0.19
ΔV * $\bar{n}_s$ * DA	0.02	-0.04 – 0.09	0.56
ΔV * $\bar{V}$ * DA	0.04	-0.03 – 0.12	0.30
$\bar{n}_s$ * $\bar{V}$ * DA	0.01	-0.04 – 0.07	0.73
ΔV * $\bar{n}_s$ * MPH	-0.02	-0.13 – 0.09	0.73
ΔV * $\bar{n}_s$ * SUL	-0.09	-0.18 – 0.00	0.47
ΔV * $\bar{V}$ * MPH	0.11	-0.01 – 0.24	0.084
ΔV * $\bar{V}$ * SUL	0.01	-0.10 – 0.14	0.88
$\bar{n}_s$ * $\bar{V}$ * MPH	0.08	-0.00 – 0.16	0.053
$\bar{n}_s$ * $\bar{V}$ * SUL	0.07	0.00 – 0.15	0.061
ΔV * DA * MPH	0.03	-0.13 – 0.17	0.71
ΔV * DA * SUL	-0.05	-0.17 – 0.06	0.40
ΔV * $\bar{n}_s$ * MPH	0.03	-0.04 – 0.10	0.41
ΔV * $\bar{n}_s$ * SUL	-0.05	-0.13 – 0.01	0.16
$\bar{V}$ * DA * MPH	0.04	-0.03 – 0.13	0.33
$\bar{V}$ * DA * SUL	-0.00	-0.09 – 0.08	0.93
$\bar{V}$ * DA * Δn_s	0.04	-0.02 – 0.11	0.23
$\bar{V}$ * MPH * Δn_s	-0.02	-0.13 – 0.09	0.73
$\bar{V}$ * SUL * Δn_s	-0.02	-0.12 – 0.08	0.71
DA * MPH * Δn_s	-0.09	-0.20 – 0.03	0.13
DA * SUL * Δn_s	-0.05	-0.16 – 0.07	0.40
ΔV * $\bar{n}_s$ * $\bar{V}$ * DA	-0.05	-0.12 – 0.02	0.16
ΔV * $\bar{n}_s$ * $\bar{V}$ * MPH	-0.01	-0.10 – 0.08	0.84
ΔV * $\bar{n}_s$ * $\bar{V}$ * SUL	-0.07	-0.15 – 0.02	0.11
ΔV * $\bar{n}_s$ * DA * MPH	-0.02	-0.10 – 0.06	0.64
ΔV * $\bar{n}_s$ * DA * SUL	-0.01	-0.10 – 0.08	0.84
ΔV * $\bar{V}$ * DA * MPH	0.01	-0.12 – 0.12	0.88

$\Delta V * \overline{V} * DA * SUL$	-0.05	-0.19 – 0.08	0.48
$\overline{n}_s * \overline{V} * DA * MPH$	0.01	-0.09 – 0.09	0.84
$\overline{n}_s * \overline{V} * DA * SUL$	-0.05	-0.14 – 0.03	0.25
$\overline{V} * DA * MPH * \Delta n_s$	0.00	-0.12 – 0.12	0.99
$\overline{V} * DA * SUL * \Delta n_s$	-0.09	-0.20 – 0.01	0.093
$\Delta V * \overline{n}_s * \overline{V} * DA * MPH$	0.07	-0.04 – 0.17	0.19
$\Delta V * \overline{n}_s * \overline{V} * DA * SUL$	0.05	-0.06 – 0.15	0.36
N_{id}	81		
Observations	24522		

Effects of dopamine factors on RL model parameters

Linear mixed effects models were fitted (using *lmer* version 3.1-3 in R) to estimate the effects of dopamine synthesis capacity on RLWM model parameters. Specifically, we examined relationships between dopamine factors and three parameters: WM reliance (ρ), the learning rate (α_{RL}), and working memory capacity (WM_{cap}) because of prior work linking individual differences to various working memory capacity measures (e.g. [1,2]). The tables below report the fitted models for those three including the corrected and uncorrected p-values after Bonferroni correction for three tests.

Supplementary Table S3

	Effect on z-scored working memory reliance (ρ)			
Predictors	*Estimate*	*CI*	*p*	$p_{corrected}$
Intercept	0.066	-0.14 – 0.27	0.53	-
DA Synth. Capacity (DA)	0.23	0.027 – 0.43	**0.029**	.087
Methylphenidate (MPH)	0.083	-0.15 – 0.31	0.48	-
Sulpiride (SUL)	-0.35	-0.57 – -0.13	**0.0028**	**0.0084**
Session	0.075	-0.019 – 0.19	0.12	0.48
DA * MPH	-0.20	-0.43 – 0.021	0.078	0.23
DA * SUL	0.11	-0.12 – 0.33	0.36	-
N_{id}	92			
Observations	249			
Random Effects (Participant):	*Variance*	*Std. Dev.*	*Corr.*	
Intercept	0.42	0.65		
Session	0.065	0.25	0.09	
Random Effects (Residual):	0.45	0.67		

Supplementary Table S4

	Effect on z-scored RL learning rate (α_{RL})			
Predictors	*Estimate*	*CI*	*p*	$p_{corrected}$
Intercept	-0.0057	-0.22 – 0.21	0.99	-
DA Synth. Capacity (DA)	-0.027	-0.24 – 0.18	0.80	-
Methylphenidate (MPH)	0.15	-0.12 – 0.42	0.29	0.87
Sulpiride (SUL)	-0.10	-0.37 – 0.17	0.46	-
Session	0.042	-0.078 – 0.16	0.49	-
DA * MPH	0.30	0.029 – 0.57	**0.032**	0.096
DA * SUL	0.041	-0.23 – 0.31	0.76	-
N_{id}	92			
Observations	249			
Random Effects (Participant):	*Variance*	*Std. Dev.*	*Corr.*	
Intercept	0.25	0.50		
Session	0.069	0.26	0.30	
Random Effects (Residual):	0.68	0.82		

Supplementary Table S5

	Effect on z-scored WM capacity (WM_{cap})			
Predictors	*Estimate*	*CI*	*p*	$p_{corrected}$
Intercept	-0.072	-0.28 – 0.14	0.50	-
DA Synth. Capacity (DA)	-0.021	-0.41 – -0.0012	0.051	0.15
Methylphenidate (MPH)	0.22	-0.054 – 0.49	0.12	0.36
Sulpiride (SUL)	-0.0086	-0.28 – 0.26	0.95	-
Session	0.034	-0.082 – 0.15	0.57	-
DA * MPH	-0.11	-0.38 – 0.16	0.43	-
DA * SUL	0.093	-0.17 – 0.36	0.49	-
N_{id}	92			
Observations	249			
Random Effects (Participant):	*Variance*	*Std. Dev.*	*Corr.*	
Intercept	0.20	0.45		
Session	0.029	0.17	-0.96	
Random Effects (Residual):	0.74	0.86		

Early versus late logistic regressions of training phase accuracy on set size, drug, and dopamine synthesis capacity

We fit a set of logistic regressions to test whether dopamine factors (drug and dopamine synthesis capacity) altered trial-wise accuracy early versus late in a session. Early in a block included all stimuli with fewer than three previous correct iterations while late in a block included the last two iterations for each stimulus. The tables below report the results of models fitted using *lmer* version 3.1-3 in R and the binomial logit link function.

The first table shows the model fitted to early trials.

Supplementary Table S6

	**Effect on trial wise accuracy in early trials**		
Predictors	*Estimate (logit)*	*CI*	*p*
Intercept	2.66	2.49 – 2.82	< **$2.1*10^{-16}$**
Set Size (n_s)	-0.45	-0.54 – -0.36	< **$2.1*10^{-16}$**
DA Synth. Capacity (DA)	0.14	0.023 – 0.26	**0.019**
Methylphenidate (MPH)	0.12	-0.073 – 0.30	0.23
Sulpiride (SUL)	-0.20	-0.38 – -0.027	**0.024**
N_{id}	92		
Observations	21475		
Random Effects (Participant):	*Variance*	*Std. Dev.*	*Corr.*
Intercept	0.37	0.61	
n_s	0.087	0.30	-0.17
MPH	0.34	0.59	-0.42
SUL	0.32	0.57	-0.54

The next table shows the model fitted to late trials in each block.

Supplementary Table S7

	Effect on trial wise accuracy in late trials		
Predictors	*Estimate (logit)*	*CI*	*p*
Intercept	3.79	3.56 – 4.02	< **$2.1*10^{-16}$**
Set Size (n_s)	-0.016	-0.13 – 0.10	0.79
DA Synth. Capacity (DA)	0.12	-0.053 – 0.29	0.18
Methylphenidate (MPH)	0.48	0.15 – 0.80	**0.0038**
Sulpiride (SUL)	-0.35	-0.61 – -0.094	**0.0072**
N_{id}	92		
Observations	19537		
Random Effects (Participant):	*Variance*	*Std. Dev.*	*Corr.*
Intercept	0.46	0.68	
n_s	0.087	0.30	0.20
MPH	0.45	0.67	-0.21
SUL	0.21	0.46	-0.20

The next table shows the model fitted to trials either early or late in each block. Note that we did not model the full covariance matrix of the random effects because model estimation failed to converge if so.

Supplementary Table S8

	**Effect on trial wise accuracy in early or late trials**		
Predictors	*Estimate (logit)*	*CI*	*p*
Intercept	2.63	2.46 – 2.76	$<$ **$2.1*10^{-16}$**
Late vs. Early (LvE)	1.25	1.04 – 1.45	$<$ **$2.1*10^{-16}$**
Set Size (n_s)	-0.34	-0.41 – -0.26	$<$ **$2.1*10^{-16}$**
DA Synth. Capacity (DA)	0.14	0.029 – 0.26	**0.014**
Methylphenidate (MPH)	0.12	-0.061 – 0.30	0.20
Sulpiride (SUL)	-0.19	-0.35 – -0.027	**0.022**
LvE * DA	-0.0099	-0.15 – 0.13	0.89
LvE * MPH	0.29	0.028 – 0.54	**0.030**
LvE * SUL	-0.21	-0.43 – 0.011	0.063
N_{id}	92		
Observations	41012		
Random Effects (Participant):	*Variance*	*Std. Dev.*	*Corr.*
Intercept	0.057	0.24	-
LvE (early)	0.088	0.30	-
LvE (late)	0.36	0.60	0.63
n_s	0.080	0.28	-
PBO	0.17	0.41	-
MPH	0.24	0.49	0.26
SUL	0.13	0.36	0.17

Supplementary Methods

Learning algorithm – posterior predictive checks and parameter recoverability

A key test of model quality is whether an algorithm can recapitulate behavioral patterns. To test this, we simulated data from our fitted model (Supplementary Figure S1). The resulting simulations reveal that the model captures key features of the data including sensitivity to iteration number, set size, dopamine synthesis capacity, early versus late trials, and delay.

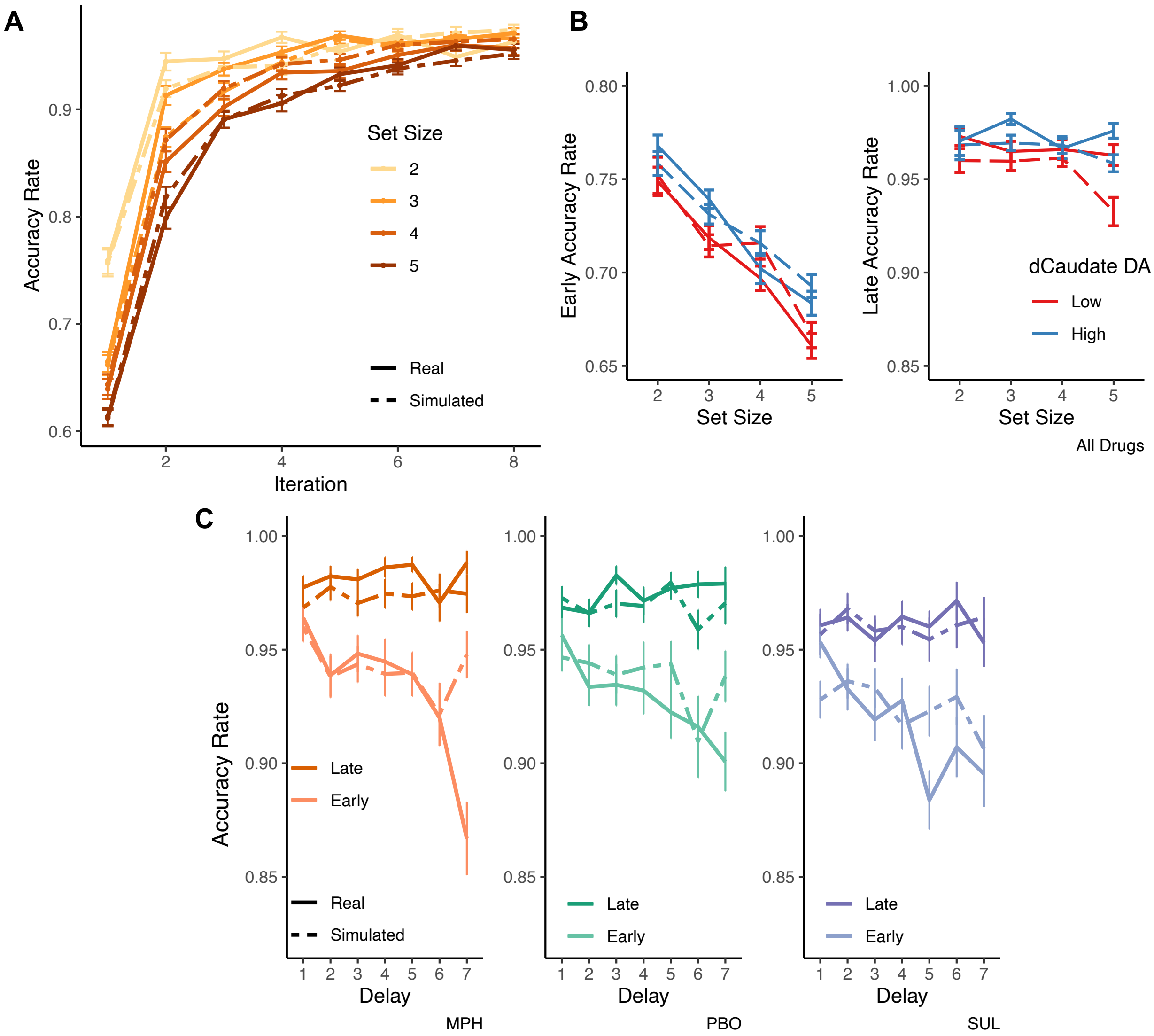

Supplementary Figure S1. Model fit to behavior. The fitted model captures key effects including **A)** iteration number and set size and **B)** dopamine synthesis capacity and set size effects which are present early but not late in a block, and **C)** drug effects across sessions as well as the effects of delay, which are present early, but not late in a block. These simulations are based on (n = 90 participants for the methylphenidate or MPH session, n = 90 participants for the placebo or PBO session, and n = 92 participants for the sulpiride or SUL session).

While our estimated parameters – the RL learning rate (α_{RL}) and the initial WM weighting (ρ), in particular – converge with model-independent analyses of behavior, the ability to draw

inferences from fitted parameters depends on whether parameter estimates are recoverable. That is, inferential capacity depends on whether one can trust that fitted parameters are an accurate reflection of their true values, given the model. One way to test this is to simulate artificial data from randomly chosen parameter value combinations (where parameters values are randomly chosen from across the range of values estimated from real data), and then fit the model to the behavior generated by these simulated agents. If the fitted values of the model match the randomly chosen value of each agent, this implies that, given the model, we can trust that parameters reliable and interpretable – i.e., that they are *recoverable*.

As revealed by the strong correlations between fitted and simulated parameter values, key parameters, including both α_{RL}, and ρ, are recoverable, with high fidelity (Supplementary Figure S2). Some parameters, e.g. the testing phase noise, appear to not be recoverable, however this did not undermine the recoverability of key terms.

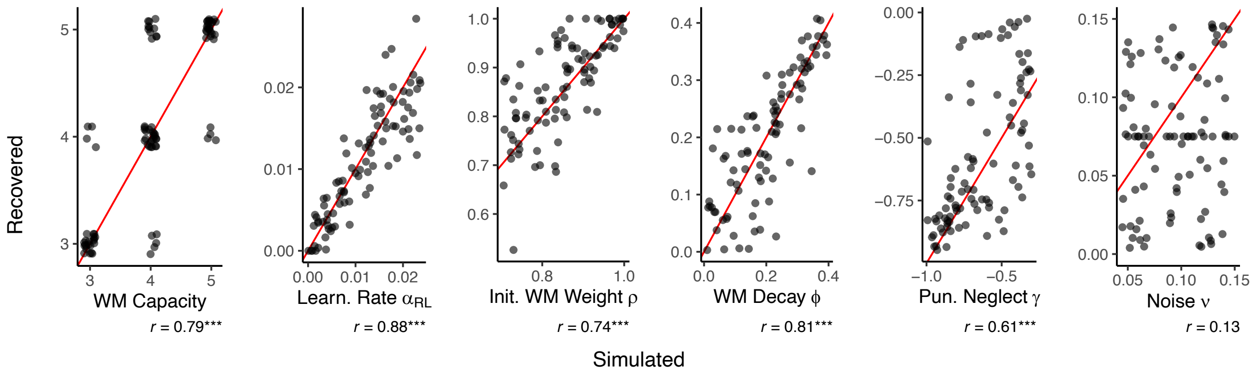

Supplementary Figure S2. A comparison of simulated and recovered parameter values for simulated agents reveals that key parameters are recoverable in our model. Data are simulated and parameters recovered for n = 92 sessions. *** indicates $p < .001$: $p < 2.1\times10^{-16}$ for WM capacity, $p < 2.1\times10^{-16}$ for the learning rate, $p < 2.1\times10^{-16}$ for initial WM weighting, $p < 2.1\times10^{-16}$ for WM decay, $p = 1.2\times10^{-10}$ for punishment neglect, and $p = .22$ for the noise term.

The distribution of fitted parameters in all three drug sessions and the criteria for outlier parameter values are shown in the following histograms.

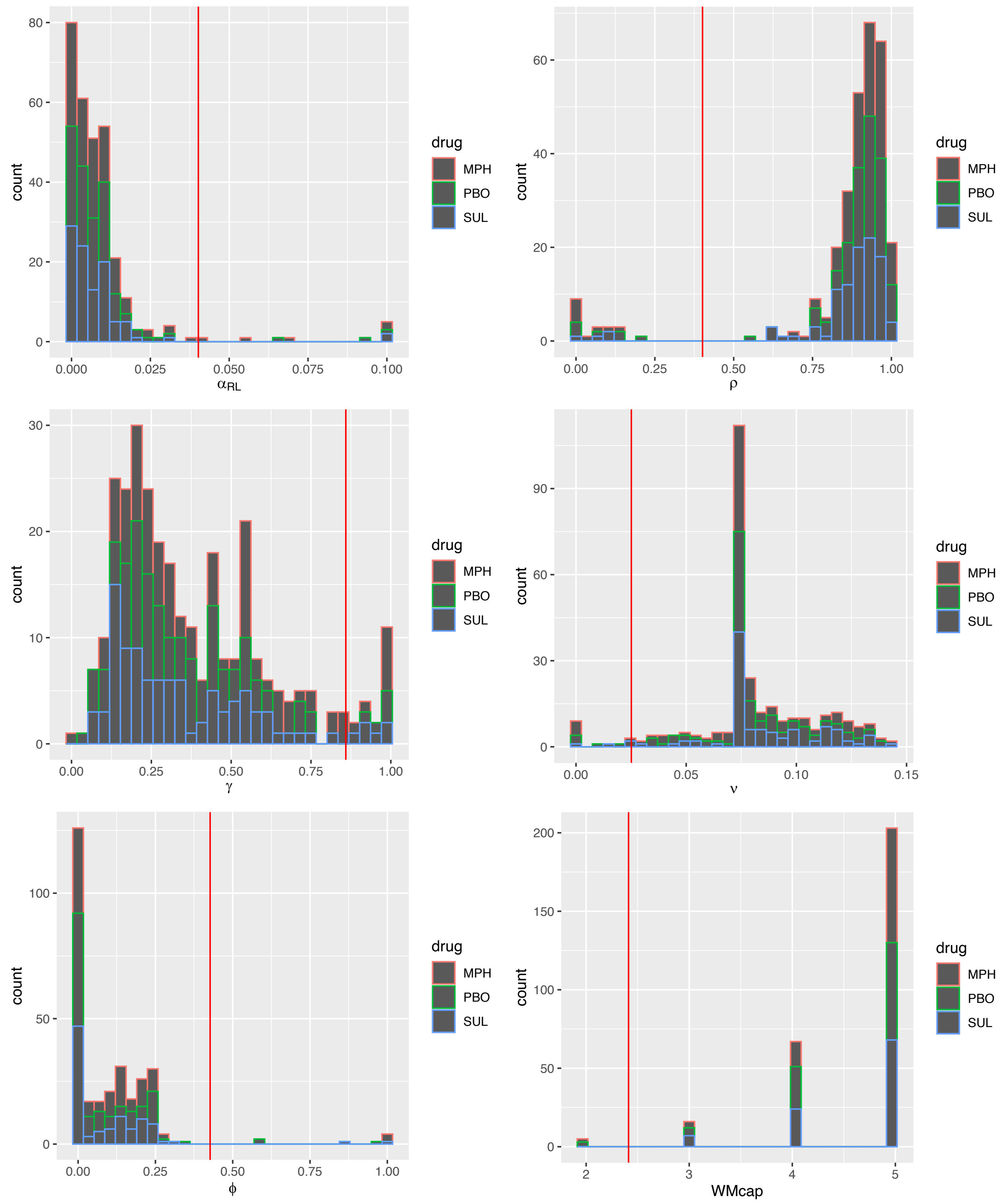

Supplementary Figure S3. The distribution of fitted parameter values in each drug session. Vertical red lines indicate outlier values based on a +/- 2 SD cutoff.

The removal of outliers did not affect any of the core inferences in the manuscript. There are some changes in exact p-values when we include sessions with outlier values for two parameters (WM decay ϕ, and WM capacity WM_{cap}). However, in neither case do the inferences which we make change fundamentally.

Regarding the WM decay term (ϕ), if we do not remove sessions with outlier value estimates, the overall positive relationship between DA synthesis capacity and WM reliance (ρ) across sessions is no longer significant (β = .16; p = .10). However, positive correlations remain between DA synthesis capacity and WM reliance in the placebo (*r* = .24; p = .028) and sulpiride sessions (*r* = .28; p = .0098). Similarly, the relationship between dopamine synthesis capacity and the learning rate is no longer significant in the methylphenidate session (β = .17; p = .13). However, a two-way interaction between dopamine synthesis capacity and methylphenidate remains, indicating that the relationship between the learning rate and dopamine synthesis capacity is stronger on methylphenidate versus placebo (β = .28; p = .032). Thus, in both cases, including outlier WM decay parameter values does not fundamentally alter the interpretation, even if there are small changes to the resulting p-values.

When we do not remove outlying WM capacity values (i.e., WM_{cap} = 2), the overall positive relationship between DA synthesis capacity and WM reliance across sessions is no longer significant (β = .19; p = .062). However, positive correlations remain between DA synthesis capacity and WM reliance in the placebo (*r* = .22; p = .041) and sulpiride sessions (*r* = .28; p = .0098) separately, indicating that not excluding on the basis of outlying WM capacity also does not fundamentally alter these inferences.

Model Selection

All the models we tested were variants of the original model proposed by [3] and [4] combining contributions of both a WM and a RL module to action selection on every trial. A key difference regards mediation between WM versus RL across trials. In the original Collins and Frank model, mediation evolves dynamically as a function of the relative confidence ascribed to each module (so that RL contributes more with increasing experience, later in each block). In the Master et al. variant, the degree to which WM contributes to a choice is fixed across each block and varies, between blocks, as a function of set size. We initially adopted the Master et al. approach because it improved parameter recoverability yet found that, sensibly, we could not capture within-block changes in the effects of delay or the difference in set size effects, early versus late in a block.

Thus, as a compromise between these approaches, we developed a new subset of variants in which the relative contribution of the WM system was set for each block, as a function of set size, but also as a function of WM load relative to capacity. Specifically, we multiplied the WM reliance parameter (ρ) by the ratio of WM capacity to the number of unique intervening stimuli since the last correct response to a given item (see Equation 5). This subset of variants had improved recoverability and also captured within-block dynamics. We examined five closely related models in this subset:

Model A. A model in which we multiply ρ by $e^{((1-n_{delay})/10)}$ where n_{delay} is the total number of trials since the last correct iteration of a stimulus.

Model B. A model in which we multiply ρ by $min\left(1, \frac{WM_{cap}}{n_{delay}}\right)$ where n_{delay} is the total number of trials since the last correct iteration of a stimulus

Model C. A model in which we multiply ρ by $min\left(1, \frac{WM_{cap}}{n_{delay,k}}\right)$ where $n_{delay,k}$ is the number of unique, correct, intervening items since the last correct iteration of a stimulus.

Model D. A model in which we multiply ρ by $min\left(1, \frac{WM_{cap}}{n_{delay,m}}\right)$ where $n_{delay,m}$ is the number of unique, intervening items since the last correct iteration of a stimulus.

Model E. A model in which we multiply ρ by $min\left(1, \frac{WM_{cap}}{n_{delay,k}}\right)$ where $n_{delay,k}$ is the number of unique, intervening items since the last correct iteration of a stimulus. In this model we also allowed for low fidelity of the WM contents. Namely, the same noise parameter which was applied to choice during the test phase (Equation 7) was also applied to choice during the training phase. Note that we originally omitted this term from the training phase on the assumption that WM fidelity is high (when load is under capacity, during a given block). We considered adding the noise parameter back, in this model, because we wanted to know how it would interact with a dynamically varying contribution of working memory to choice, during training.

Although BIC scores were very similar across this subset of model variants, as shown in Figure S4, the winning model, *Model C* (in red) was slightly better for a majority of participants and drug sessions. In pairwise comparisons, *Model C* beat *Model A* for 93.1% of sessions, *Model B* for 87.3% of sessions, *Model D* for 52.2% of sessions, and *Model E* for 63.2% of sessions.

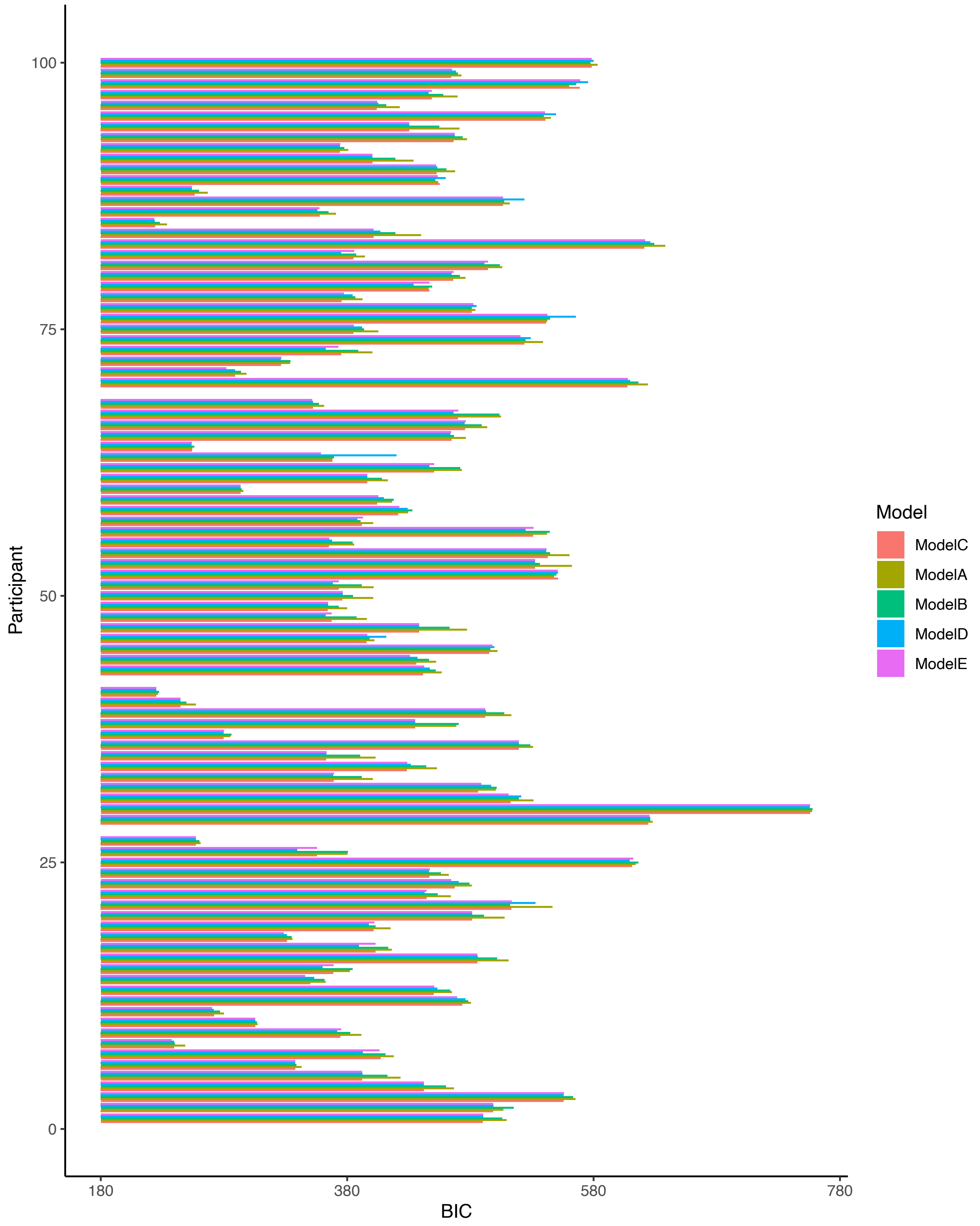

Supplementary Figure S4. BIC scores for each participant in the placebo session, for five model variants.

Description of the dorsal caudate nucleus used for PET analyses

For our analyses of the effect of individual differences in dopamine synthesis capacity, we used the dorsal caudate nucleus (green region in Supplementary Figure S5) as independently defined from a study which partitioned the striatum based on cortical connectivity patterns [5]. As noted, the dorsal caudate nucleus was chosen based on prior work linking this region to higher order cognitive regions and cognitive function (e.g. including the dorsolateral prefrontal cortex [6]) and work implicating the dorsal caudate nucleus, in particular, in higher-order RL processes (e.g. [7]) and working memory gating (e.g. [8]).

The dorsal caudate nucleus comprises 351 voxels in 2x2x2 mm MNI space, from which dopamine synthesis capacity values were extracted.

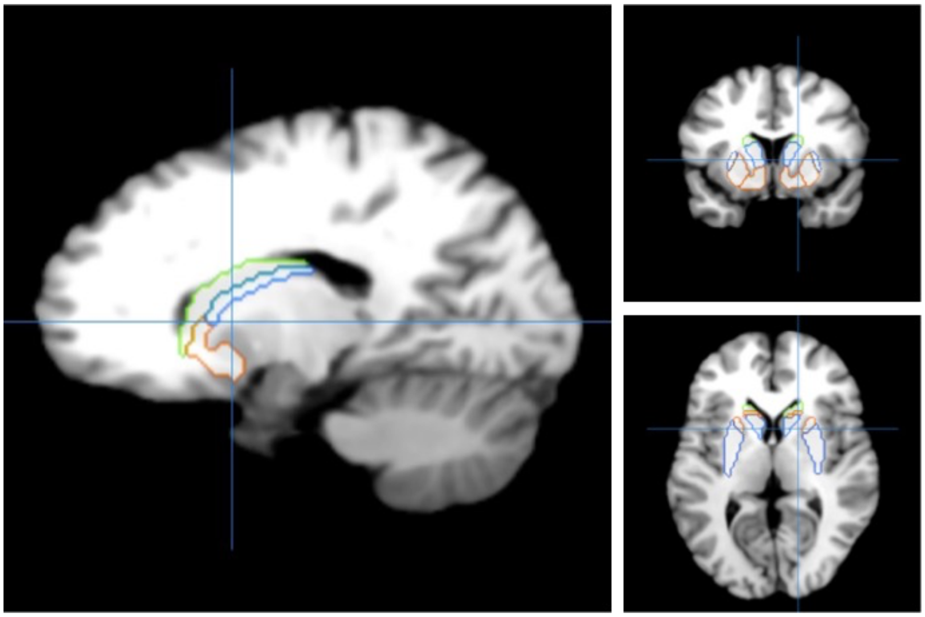

Supplementary Figure S5. Striatal subdivisions from [5] including the dorsal caudate nucleus (green region) used in this study.

Although we focused on the dorsal caudate nucleus in our analyses, we note that dopamine synthesis capacity is highly correlated across sub-regions. Thus, as an exploratory analysis, we also tested whether our model parameters related to dopamine synthesis capacity in other regions. As expected, given that dopamine synthesis capacity values are highly correlated across the striatum, we also find a significant correlation between our WM reliance parameter, ρ, and dopamine synthesis capacity in the posterior putamen (r = 0.24, p = .029) and a trending relationship in the medial caudate nucleus (r = 0.18, p = .091), but no other region (all other p's > 0.21). Also, just like our dorsal caudate nucleus region, the RL learning rate (α_{RL}) is predicted by a significant interaction between the dopamine synthesis capacity and methylphenidate in the posterior putamen (β = .28; p = .046) and the ventral striatum (β = .28; p = .047; all other p's > 0.12).

Methylphenidate does not alter test phase performance

One potential explanation for the effect of methylphenidate on performance during the test phase – when participants were asked to identify which, in each pair of pseudo-randomly

selected items, were most rewarded during the learning phased – is that methylphenidate altered long-term memory for reward experiences associated with each item. Thus, perhaps the drug effect which we interpret as an effort-discounting of experienced rewards (where rewards earned in the context of high working memory loads are subjectively less rewarding) instead reflects differences in the fidelity of memory about reward experiences, as function of load, under methylphenidate versus placebo.

To address this alternative explanation, we tested whether methylphenidate altered overall accuracy (the rate at which participants correctly identified the most rewarded item) and whether methylphenidate interacted with the difference in objective reward rates (total points earned for each item). We found that there is in fact neither a difference in terms of test phase accuracy on the methylphenidate versus placebo condition, nor a drug by value difference interaction (both p's > 0.57).

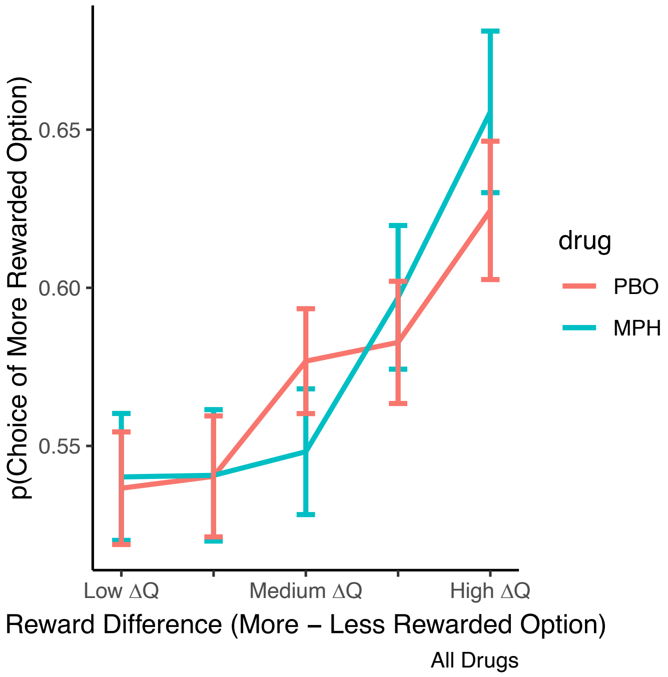

Supplementary Figure S6. No effect of drug or drug by value difference interaction on the probability that, during the test phase, participants correctly chose the most rewarded item in each pair, selected from the learning phase. Here Q value refers to the difference in objective points awarded for each item, and the difference in Q values between each item is divided into quintiles for visualization purposes. Values above 0.5 and a positive slope both indicate that participants were implicitly tracking reward statistics.

Supplementary References

1. Cools, R. (2008) Role of Dopamine in the Motivational and Cognitive Control of Behavior. *The Neuroscientist* 14, 381–395

2. Schaaf, M.E. van der *et al.* (2012) Establishing the Dopamine Dependency of Human Striatal Signals During Reward and Punishment Reversal Learning. *Cereb Cortex* 24, 633–642

3. Collins, A.G.E. and Frank, M.J. (2012) How much of reinforcement learning is working memory, not reinforcement learning? A behavioral, computational, and neurogenetic analysis. *European Journal of Neuroscience* 35, 1024–1035

4. Master, S.L. *et al.* (2020) Disentangling the systems contributing to changes in learning during adolescence. *Dev Cogn Neuros* 41, 100732

5. Piray, P. *et al.* (2017) Dopaminergic Modulation of the Functional Ventrodorsal Architecture of the Human Striatum. *Cereb Cortex* 27, 485–495

6. Haber, S.N. and Knutson, B. (2009) The Reward Circuit: Linking Primate Anatomy and Human Imaging. *Neuropsychopharmacology* 35, 4–26

7. Badre, D. and Frank, M.J. (2012) Mechanisms of Hierarchical Reinforcement Learning in Cortico-Striatal Circuits 2: Evidence from fMRI. *Cerebral Cortex* 22, 527–536

8. Fallon, S.J. *et al.* (2017) The Neurocognitive Cost of Enhancing Cognition with Methylphenidate: Improved Distractor Resistance but Impaired Updating. *J Cognitive Neurosci* 29, 652–663
