# Supplementary material for: Striatal dopamine can enhance both fast working memory, and slow reinforcement learning, while reducing implicit effort cost sensitivity
Source: Nat Commun. 2025 Jul 9;16:6320. doi: 10.1038/s41467-025-61099-0 (PMC12241355; doi:10.1038/s41467-025-61099-0)
Supplement: Supplementary file 2 — Reporting Summary [file 41467_2025_61099_MOESM2_ESM.pdf]

nature portfolio

Corresponding author(s): Westbrook, Andrew

Last updated by author(s): Mar 30, 2025

Reporting Summary

Nature Portfolio wishes to improve the reproducibility of the work that we publish. This form provides structure for consistency and transparency in reporting. For further information on Nature Portfolio policies, see our Editorial Policies and the Editorial Policy Checklist.

Statistics

For all statistical analyses, confirm that the following items are present in the figure legend, table legend, main text, or Methods section.

n/a	Confirmed	
☐	☒	The exact sample size (*n*) for each experimental group/condition, given as a discrete number and unit of measurement
☐	☒	A statement on whether measurements were taken from distinct samples or whether the same sample was measured repeatedly
☐	☒	The statistical test(s) used AND whether they are one- or two-sided<br>*Only common tests should be described solely by name; describe more complex techniques in the Methods section.*
☐	☒	A description of all covariates tested
☐	☒	A description of any assumptions or corrections, such as tests of normality and adjustment for multiple comparisons
☐	☒	A full description of the statistical parameters including central tendency (e.g. means) or other basic estimates (e.g. regression coefficient) AND variation (e.g. standard deviation) or associated estimates of uncertainty (e.g. confidence intervals)
☐	☒	For null hypothesis testing, the test statistic (e.g. *F*, *t*, *r*) with confidence intervals, effect sizes, degrees of freedom and *P* value noted<br>*Give P values as exact values whenever suitable.*
☐	☒	For Bayesian analysis, information on the choice of priors and Markov chain Monte Carlo settings
☐	☒	For hierarchical and complex designs, identification of the appropriate level for tests and full reporting of outcomes
☐	☒	Estimates of effect sizes (e.g. Cohen's *d*, Pearson's *r*), indicating how they were calculated

Our web collection on statistics for biologists contains articles on many of the points above.

Software and code

Policy information about availability of computer code

Data collection

Matlab (2018b), Psychtoolbox-3.0.12

Data analysis

R (4.3.1), Matlab (2020b)
Statistical Parametric Mapping 12 (SPM12; https://www.fil.ion.ucl.ac.uk/spm/software/spm12/)
FSL (version 6.0.1)
`dplyr` R package (2.3.4)
`ggplot2` R package (3.4.3)
`lme4` R package (1.1-35.1)
`lmerTest` R package (3.1-3)
`brms` R package (2.20.4)
`gridExtra` R package (2.3)
`ggnewscale` R package (0.4.9)
`ggsignif` R package (0.6.4)
`tidybayes` R package (3.0.6)
`ggpubr` R package (0.6.0)
`rstatix` R package (0.7.2)
`dplyr` R package (2.3.4)

For manuscripts utilizing custom algorithms or software that are central to the research but not yet described in published literature, software must be made available to editors and reviewers. We strongly encourage code deposition in a community repository (e.g. GitHub). See the Nature Portfolio guidelines for submitting code & software for further information.

Data

Policy information about availability of data

All manuscripts must include a data availability statement. This statement should provide the following information, where applicable:

- Accession codes, unique identifiers, or web links for publicly available datasets
- A description of any restrictions on data availability
- For clinical datasets or third party data, please ensure that the statement adheres to our policy

The datasets generated during and/or analyzed during the current study are available in the Radboud Data Repository: https://doi.org/10.34973/0apx-ck49

Research involving human participants, their data, or biological material

Policy information about studies with human participants or human data. See also policy information about sex, gender (identity/presentation), and sexual orientation and race, ethnicity and racism.

Reporting on sex and gender	Our recruiting was designed to balance to achieve gender balance in our final sample so that results could be generalized across men and women. Our final recruited sample include 50 women and 50 men.
Reporting on race, ethnicity, or other socially relevant groupings	Our data and analyses did not include socioeconomic variables. Our sample included volunteers from the Radboud University community and the majority of our participants were Dutch university students, from Nijmegen and surrounding areas. We did not collect demographic data beyond age and gender.
Population characteristics	One hundred healthy volunteers, 50 women and 50 men, were recruited for the study (age at inclusion: range 18 to 43, mean (SD) = 23.0 (5.0) years). All participants had Dutch as their native language and were right-handed. Exclusion criteria included any current or previous psychiatric or neurological disorders, having a first-degree family member with a current or previous psychiatric disorder, clinically significant hepatic, cardiac, renal, metabolic or pulmonary disease, epilepsy, hyper or hypotension, habitual smoking or drug use, pregnancy, and MRI contraindications, such as unremovable metal parts in the body and claustrophobia.
Recruitment	People were recruited via an advertisement on the Radboud University electronic database for research participants, and via advertisement flyers around Nijmegen. The majority of participants in the electronic database are higher education students from around Nijmegen. In combination with the fact that this was a large multi-session study to participate in, this may have caused a selection bias towards motivated participants (which may relate to dopamine levels).
Ethics oversight	This study was approved by the local ethics committee ("Commissiee Mensgebonden Onderzoek", CMO region Arhem/ Nijmegen, The Netherlands: protocol NL57538.091.16). All participants provided written, informed consent. Subjects will be paid approximately 319 Euros in total (maximum 321 Euros). This total amount will comprise 100 Euro as for the entire PET session (cognitive task, baseline measures, capsules, injection and scanning), as well as 10 Euros per hour for the fMRI session and 8 Euros per hour for behavioural tasks/questionnaires, 10 Euros per drug session for capsule intake (i.e., 3 sessions – MPH/SUL/placebo), 10 Euros for submitting the saliva sample (paid by the Human Genomics project), and an additional payment related to performance on the cognitive tasks (maximum 2 Euros).

Note that full information on the approval of the study protocol must also be provided in the manuscript.

Field-specific reporting

Please select the one below that is the best fit for your research. If you are not sure, read the appropriate sections before making your selection.

☐ Life sciences ☒ Behavioural & social sciences ☐ Ecological, evolutionary & environmental sciences

For a reference copy of the document with all sections, see nature.com/documents/nr-reporting-summary-flat.pdf

Behavioural & social sciences study design

All studies must disclose on these points even when the disclosure is negative.

Study description	Quantitative, within-subject, placebo-controlled, double-blind cross-over design
Research sample	Representative sample of 100 healthy human participants, 50% male, 50% female. Age: range 18 to 43, mean (SD) 23.0 (5.0) years. The sample size in the current work was determined by the number of participants included in the overarching project that this study is part of, rather than with an a priori power calculation for the specific task reported here.
Sampling strategy	Our sample size estimation of the overarching project that the current experiment is part of was calculated based on the effect size of a previous pharmacological-behavioral study recently performed by our group (CMO Arnhem-Nijmegen protocol 2013/568). in that study, 95 participants received placebo and methylphenidate on two sessions and performed a series of cognitive tasks. The effect size of that study was r=0.30 (p<0.001, rank correction). Here we used G*power3 software for power calculation, which demonstrated that using a multiple linear regression model with 7 predictor variables (7 task outcomes in overarching project) an

	effect-size of f2 = 0.1 (multple regression equivalent of r=0.3) may be detected with 85% power from a sample size of 92 subjects (pre-defined p value = 5%). We rounded thus number up to 100 to account for potential drop-outs and technical problems. Gender and age were recorded, and these data are available in the repository for disaggregation. However, gender was not part of any of our core hypotheses and was not analyzed as a variable of interest. Nevertheless, we conducted a follow-up analysis and confirmed that gender was not related to performance during the primary training phase of the study, confirming our decision not to include gender as a variable in core analyses.
Data collection	Task response data were collected on a computer (Windows 7 Enterprise OS), in a behavioral testing suite. PET data were acquired on a state-of-the-art PET/CT scanner (Siemens Biograph mCT; Siemens medical Systems, Erlangen, Germany). During the PET scan, medical personnel were present to administer the radiotracer and operate the PET scanner. Drug administration was double-blind and only unblinded to the expreimenters after completion of all data collection.
Timing	January 2017 - October 2018
Data exclusions	The experiment included two phases: a training phase and a test phase. From the test phase, one participants' data was excluded from analysis because their average was below 53% in all three sessions. A single session from another participant was excluded because they did not complete the training phase. In addition, three participants did not participate in the methylphenidate session, and one participant did not complete their placebo session. In total, 95 out of 100 methylphenidate, 97 out of 100 placebo, and 99 out of 100 sulpiride sessions were included in the final analysis of the training phase. In the test phase, an error with response logging meant that some sessions were excluded based on the criteria that we failed to capture participants' responses on at least 80% of trials. Additionally, two participants were excluded based on their choice patterns which indicated that they merely alternated left / right presses on more than 75% of trials. In total, 69 out of 100 methylphenidate, 78 out of 100 placebo, and 80 out of 100 sulpiride sessions were included in the final analysis of the test phase.
Non-participation	Six participants dropped out because of discomfort in the MRI or PET scanner (N = 4), personal reasons (N = 1) or technical failure of the PET scanner (N = 1).
Randomization	Participants were randomly assigned the order in which they received the two drugs and placebo.

Reporting for specific materials, systems and methods

We require information from authors about some types of materials, experimental systems and methods used in many studies. Here, indicate whether each material, system or method listed is relevant to your study. If you are not sure if a list item applies to your research, read the appropriate section before selecting a response.

Materials & experimental systems

n/a	Involved in the study	
☒	☐	Antibodies
☒	☐	Eukaryotic cell lines
☒	☐	Palaeontology and archaeology
☒	☐	Animals and other organisms
☐	☒	Clinical data
☒	☐	Dual use research of concern
☒	☐	Plants

Methods

n/a	Involved in the study	
☒	☐	ChIP-seq
☒	☐	Flow cytometry
☐	☒	MRI-based neuroimaging

Clinical data

Policy information about clinical studies
All manuscripts should comply with the ICMJE guidelines for publication of clinical research and a completed CONSORT checklist must be included with all submissions.

Clinical trial registration	https://onderzoekmetmensen.nl/en/trial/28994
Study protocol	https://osf.io/preprints/osf/d3h8e
Data collection	All data were collected at the Donders Institute and Radboud University in Nijmegen between January 2017 and October 2018.
Outcomes	Primary and secondary outcome measures including response accuracy, and selection according to reward rates were adopted from prior studies using the same Reinforcement Learning Working Memory (RLWM) task, originally described in Collins and Frank (2012) Euro. Jour. of Neurosciences (https://onlinelibrary.wiley.com/doi/full/10.1111/j.1460-9568.2011.07980.x)

Plants

Seed stocks: NA

Novel plant genotypes: NA

Authentication: NA

Magnetic resonance imaging

Experimental design

Design type: Structural imaging

Design specifications: NA

Behavioral performance measures: NA

Acquisition

Imaging type(s): structural

Field strength: 3 Tesla

Sequence & imaging parameters: T1-weighted magnetization prepared, rapid-acquisition gradient echo sequence (192 sagittal slices; repetition time, 2300 ms; echo time, 3.03 ms; FoV, 256x256 matrix; 1.0 mm in-plane resolution; 1.0 mm slice thickness)

Area of acquisition: whole brain

Diffusion MRI ☐ Used ☐ Not used

Preprocessing

Preprocessing software: NA

Normalization: NA

Normalization template: NA

Noise and artifact removal: NA

Volume censoring: NA

Statistical modeling & inference

Model type and settings: NA

Effect(s) tested: NA

Specify type of analysis: ☐ Whole brain ☒ ROI-based ☐ Both

Anatomical location(s): Dorsal and ventral caudate nucleus, putamen, and nucleus accumbens ROI masks, based on an independent, functional-connectivity-based parcellation of the striatum (Piray et al., 2017. Cerebral Cortex) were used for extracting PET values.

Statistic type for inference: NA
(See Eklund et al. 2016)

Correction: NA

Models & analysis

n/a	Involved in the study
☒	☐ Functional and/or effective connectivity
☒	☐ Graph analysis
☒	☐ Multivariate modeling or predictive analysis
